# Clinical and Pathologic Characterization of Proteinuric Kidney Disease in Australian and New Zealand Dogs

**DOI:** 10.1111/jvim.70187

**Published:** 2025-07-21

**Authors:** Lucy Kopecny, Joanna D. White

**Affiliations:** ^1^ Small Animal Specialist Hospital North Ryde North Ryde New South Wales Australia

**Keywords:** canine, glomerular disease, protein losing nephropathy, renal biopsy

## Abstract

**Background:**

The prevalence of immune complex‐mediated glomerulonephropathy (ICGN) in dogs with proteinuric kidney disease is approximately 50% in the United States and Europe but is unknown in other locations such as Australia and New Zealand.

**Objectives:**

Determine the prevalence of ICGN in dogs biopsied for proteinuric kidney disease in Australia and New Zealand and compare clinicopathologic variables in dogs with specific pathologic lesions.

**Animals:**

Fifty client‐owned dogs.

**Methods:**

Retrospective case series. Reports from renal biopsy samples submitted to the Texas and International Veterinary Renal Pathology Services from dogs with proteinuric kidney disease (urine protein‐to‐creatinine ratio ≥ 0.5) between 2007 and 2023 were reviewed. Clinical data were retrieved and compared.

**Results:**

Among 50 dogs with proteinuric renal disease, 15 dogs (30%) had ICGN and 35 (70%) had non‐ICGN. The most common category of ICGN was membranous glomerulonephropathy (6/15; 40%). Glomerulosclerosis was the most common category of non‐ICGN (17/35; 49%). Dogs with glomerulosclerosis (median, 10 years) were older than dogs with other types of lesions (membranoproliferative, mesangioproliferative or mixed pattern; median, 6 years; *p* = 0.04) and those with membranous glomerulonephropathy (median, 4 years; *p* = 0.005). Dogs with membranous glomerulonephropathy had lower serum albumin concentrations (median, 2.1 g/dL) than dogs with glomerulosclerosis (median, 3.0 g/dL; *p* = 0.01) or other nephropathies (median, 3.0 g/dL; *p* = 0.04).

**Conclusions and Clinical Importance:**

The prevalence of ICGN is lower in dogs in Australia and New Zealand biopsied for proteinuric kidney disease, potentially because of a lower prevalence of infectious disease, particularly vector‐borne disease. The lower prevalence of ICGN emphasizes the importance of renal biopsy to optimize treatment.

**Abbreviations**
ICGN: immune complex‐mediated glomerulonephropathy
RR: reference range
UPCR: urine protein‐to‐creatinine ratio

## 1 Introduction

Glomerular disease, which can be immune or non‐immune complex‐mediated, is recognized in dogs worldwide. Understanding the prevalence of glomerular diseases in different regions could provide epidemiological evidence to clarify the underlying etiopathogeneses. At a regional level, disease prevalence also informs the pre‐test probability of specific glomerular diseases during investigations such as renal biopsy and can guide treatment while diagnostic test results are pending.

Renal biopsy samples from dogs in Europe, predominantly from Sweden, the United Kingdom, and the Netherlands, determined that 50% of dogs biopsied had immune complex‐mediated glomerulonephropathy (ICGN) [1]. This finding is similar to results from North America, where 48% of dogs biopsied for suspected glomerular disease had ICGN [2]. In contrast, renal biopsy samples from dogs in the United Kingdom with persistent proteinuria showed only 27% had ICGN [3]. It was hypothesized that this difference could be associated with a lower prevalence of infectious disease in the United Kingdom compared with North America and Europe [3].

Australia and New Zealand have a lower prevalence of infectious diseases, particularly vector‐borne diseases, which are associated with some forms of ICGN [4]. Infection by *Borrelia burgdorferi*, an important cause of membranoproliferative glomerulonephritis, is not recognized in Australia and New Zealand [4, 5]. Similarly, Australia is considered free from *Leishmania infantum*, which causes ICGN and tubulointerstitial inflammation [4, 6]. Until recently, *Ehrlichia canis* was exotic to Australia, and is still mostly reported in specific regions in northern Australia [7]. *Anaplasma platys* also appears to be restricted to certain areas of Australia, such as free‐ranging dogs near Indigenous communities in central Australia and is less common in similar communities in northwestern New South Wales [8]. *Babesia vogeli* is endemic in parts of Queensland and the Northern Territory [9, 10], whereas *Babesia gibsoni* has only been detected in Australia in American Pit Bulls and likely is transmitted blood‐to‐blood by dog bites [11, 12]. The prevalence of heartworm disease is low and also generally isolated to specific areas [13].

We hypothesized that the prevalence of ICGN would be lower in Australian and New Zealand dogs than in dogs in North America and Europe. Therefore, our primary aim was to determine the prevalence of ICGN in dogs biopsied for evaluation of proteinuric kidney disease in Australia and New Zealand. A secondary aim was to evaluate the associations of clinicopathologic variables with specific glomerular lesions.

## 2 Materials and Methods

### 2.1 Case Selection Criteria and Medical Records Review

The International Veterinary Renal Pathology Service database was searched for renal biopsy samples in dogs from Australia and New Zealand. Dogs were eligible for inclusion in the study if they had undergone a renal biopsy for investigation of proteinuric kidney disease where the renal biopsy sample was evaluated by light microscopy and where indicated, by immunofluorescence and transmission electron microscopy. Proteinuric kidney disease was defined as urine protein‐to‐creatinine ratio (UPCR) persistently ≥ 0.5 [14].

The medical records of included dogs were reviewed and information on breed, sex, neuter status, age, and blood pressure at the time of renal biopsy was recorded for each dog. Any available recent drug history also was included. Clinicopathologic data collected included serum creatinine and albumin concentrations and UPCR before renal biopsy.

### 2.2 Renal Biopsy

Renal biopsy samples submitted to the Texas Veterinary Renal Pathology Service or International Veterinary Renal Pathology Service were categorized by the nephropathologist using established pathological criteria [15, 16]. Based on the renal biopsy report, glomerular disease was characterized as ICGN or non‐ICGN. Immune complex‐mediated glomerulonephropathy included dogs with glomerulonephritis and membranous glomerulonephropathy.

Dogs were further categorized using the renal biopsy report as:

- Glomerulonephritis including dogs with membranoproliferative glomerulonephritis, mesangioproliferative glomerulonephritis, and mixed immune complex–mediated glomerulonephritis.
- Membranous glomerulonephropathy.
- Glomerulosclerosis including dogs with focal segmental or global glomerulosclerosis.
- Other nephropathies including primary tubular and interstitial lesions with secondary glomerular changes, juvenile nephropathy, and acute tubular injury [15, 16].

### 2.3 Statistical Analysis

Descriptive data were generated for clinicopathological data stratified by pathology. Continuous variables were assessed for normality using the Shapiro–Wilk test and described by mean and SD and median and quartiles for parametric and non‐parametric variables, respectively. Differences in variables including age, sex, UPCR, and serum creatinine and albumin concentrations among pathological groups were compared using Kruskal–Wallis rank sum test (continuous variables) and Fisher's exact test (categorical variables). Where a significant difference was identified among all groups, subsequent pairwise comparisons were corrected for multiple comparisons using the Benjamini–Hochberg procedure (false discovery rate) and adjusted *p* values were assessed. The single dog with amyloidosis was removed for these comparisons because there was only one dog in this disease category.

## 3 Results

Renal biopsy samples from 50 dogs with proteinuric kidney disease from Australia and New Zealand were included between January 2007 and December 2023. Renal biopsy samples from 11 dogs with non‐proteinuric kidney disease were excluded. Renal biopsy samples were submitted from veterinary hospitals in New South Wales (39/50; 78%), Western Australia (6/50; 12%), South Australia (4/50; 8%), and New Zealand (1/50; 2%).

The median age at the time of biopsy was 6.0 (quartile 1–quartile 3 [Q1–Q3], 4.0–10.0) years. Of 50 dogs, 25 (50%) were neutered males, 17 (34%) spayed females, 5 (10%) intact females, and 3 (6%) intact males. The most common breed was Miniature Schnauzer (10/50; 20%). Other breeds represented were Boxer (2/50; 4%), Beagle (2/50; 4%), Border Collie (2/50; 4%), Cavalier King Charles Spaniel (2/50; 4%), Golden Retriever (2/50; 4%), Labrador Retriever (2/50; 4%), and Staffordshire Bull Terrier (2/50; 4%).

Before renal biopsy, four dogs had recently received antimicrobials, with three dogs receiving amoxicillin clavulanate for urinary tract infection, tooth root abscess, and possible bacterial cholangiohepatitis, and one dog receiving doxycycline for suspected lymphoplasmacytic rhinitis. Two dogs were receiving prednisolone at the time of renal biopsy for immune‐mediated polyarthritis and steroid‐responsive meningitis‐arteritis, respectively. One dog recently was treated with mycophenolate for suspected ICGN, but this medication was discontinued because of gastrointestinal adverse effects before renal biopsy; this dog had membranous glomerulonephropathy on biopsy.

The median UPCR at the time of renal biopsy was 4.1 (Q1–Q3, 2.8–9.0; reference range [RR], < 0.2). At the time of biopsy, the median serum creatinine concentration was 2.0 (Q1–Q3, 1.1–3.4; RR, 0.8–1.5) mg/dL. The mean serum albumin concentration was 2.8 (standard deviation ±2.2; RR, 3.4–4.3) g/dL. The median systolic blood pressure was 146 (Q1–Q3, 134–170) mmHg.

Of 50 dogs, 15 (30%) had ICGN and 35 (70%) had non‐ICGN lesions. The most common category of ICGN was membranous glomerulonephropathy (6/15; 40%). Of 15 dogs with ICGN, 3 (20%) had a mixed pattern, 3 (20%) had ICGN that could not be further categorized, 2 (13%) had membranoproliferative glomerulonephritis, and 1 (7%) had mesangioproliferative glomerulonephritis.

Glomerulosclerosis was the most common category of non‐ICGN lesion (17/35; 49%). The most common category of glomerulosclerosis was focal segmental glomerulosclerosis (10/17), but many of these dogs had extension of focal segmental changes to include some global glomerulosclerosis. Of 17 dogs with glomerulosclerosis, 7 were female (41%) and 10 were male (59%). One dog had amyloidosis. Dogs with other nephropathies had juvenile nephropathy (1), interstitial nephritis (6), acute tubular injury (3), arterionephrosclerosis (3), glomerular hypertrophy (1), podocyte injury (1), tubular vacuolar degeneration (1), glomerular lipid deposits and glomerular lipid thromboemboli (1), and unclassified nephropathy (0).

Of the 10 miniature Schnauzers included, 4/10 (40%) had glomerulosclerosis and 2/10 (20%) had interstitial nephritis. One miniature Schnauzer (10%) had evidence of immune complex deposits, and 1 (10%) had glomerular lipid deposits and glomerular lipid thromboemboli.

Clinicopathological variables differed among dogs with different glomerular lesions. Overall, differences were found between pathological groups with regard to age (*p* < 0.001) and serum albumin concentration (*p* < 0.02). Specifically, after correction for pair‐wise comparisons, dogs with glomerulosclerosis (median, 10 years; Q1–Q3, 8–11 years) were older than dogs with membranoproliferative, mesangioproliferative, or a mixed pattern (median, 6 years; Q1–Q3, 5–8 years; *p* = 0.04), membranous glomerulonephropathy (median, 4 years; Q1–Q3, 2–5 years; *p* = 0.005), and other nephropathies (median, 4 years; Q1–Q3, 2.3–8.5 years; *p* = 0.005; Table 1). Dogs with membranous glomerulonephropathy had lower serum albumin concentrations (median, 2.1 g/dL; Q1–Q3, 2.0–2.1 g/dL) than dogs with either glomerulosclerosis (median, 3.0 g/dL; Q1–Q3, 2.8–3.3 g/dL; *p* = 0.01) or other nephropathies (median, 3.0 g/dL; Q1–Q3, 2.6–33.7 g/dL; *p* = 0.04; Table 1).

**TABLE 1:** Clinicopathological variables from Australian and New Zealand dogs with protein losing nephropathies (50 dogs, 2007–2023).

Pathology (number)	Age (years) median (Q1–Q3)	Sex (male:female) number	Creatinine (mg/dL) median (Q1–Q3)	UPC median (Q1–Q3)	Albumin (g/dL) median (Q1–Q3)	SBP (mmHg) median (Q1–Q3)
GN (9 dogs)	6.0^a^ (5.0–8.0)	4:5 dogs	3.5^a^ (1.9–4.7)	2.9^a^ (2.3–9.0)	2.6^a^ (1.8–3.0)	140.0 (118.8–168.5)
GS (17 dogs)	10.0^b,c^ (8.0–11.0)	10:7 dogs	1.3^a,b^ (0.9–2.0)	5.1^a,b^ (3.4–7.8)	3.0^a,b^ (2.8–3.3)	150.5 (140.0–170.0)
Membranous (5 dogs)	4.0^a,d,e^ (2.0–5.0)	3:2 dogs	1.8^a,b,d^ (1.1–1.9)	10.0^a,b,c^ (6.4–14.1)	2.1^a,c,d^ (2.0–2.1)	140.0 (115.0–145.0)
Other (18 dogs)	4.0^a,d,e^ (2.3–8.5)	10:8 dogs	3.1^a,c,d^ (1.8–3.4)	2.8^a,c,d^ (1.5–4.1)	3.0^a,b,e^ (2.6–3.7)	143.5 (137.5–160.0)
*p* value	< 0.001	0.94	0.02	0.01	0.02	0.5

*Note:* Values with different superscript letters in a column are significantly different (*p* < 0.05).

Abbreviations: GN, glomerulonephritis; GS, glomerulosclerosis; MGN, membranous glomerulopathy; SBP, systolic blood pressure; UPC, urine protein‐to‐creatinine ratio.

Overall, significant differences were found in proteinuria (*p* = 0.01) and serum creatinine concentrations (*p* = 0.02) between pathological groups. Specifically, after corrections for pair‐wise comparisons, dogs with other nephropathies had higher serum creatinine concentrations (median, 3.1 mg/dL; Q1–Q3, 1.8–3.4 mg/dL) than dogs with glomerulosclerosis (median, 1.3 mg/dL; Q1–Q3, 0.9–2.0 mg/dL; *p* = 0.03). Dogs with other nephropathies (median UPCR, 2.8; Q1–Q3, 1.5–4.1) had lesser degrees of proteinuria than dogs with either glomerulosclerosis (median UPCR, 5.1; Q1–Q3, 3.4–7.8; *p* = 0.05) or membranous glomerulonephropathy (median UPCR, 10.0; Q1–Q3, 6.4–14.1; *p* = 0.05; Table 1).

No significant difference was found in systolic blood pressure (*p* = 0.5) or sex distribution (*p* = 0.9) between dogs with different proteinuric kidney diseases (Table 1).

## 4 Discussion

Dogs in Australia and New Zealand biopsied for persistent proteinuria had a lower prevalence of ICGN than similar dogs in North America and Europe [1, 2]. The prevalence in Australia is similar to that in the United Kingdom [3]. The variation in ICGN prevalence across different countries could indirectly support an association of infectious agents, particularly those that are vector‐borne, in the pathogenesis of immune complex deposition in some forms of ICGN [4].

Vector‐borne diseases are less common in Australia and New Zealand, with those such as *A. platys* , *E. canis* , and *B. vogeli* mostly restricted to central and northern Australia [8, 9, 10]. Because fewer veterinary referral hospitals are located in these regions, dogs from these regions were unlikely to be represented in our population, with included dogs being from veterinary hospitals in western, eastern and southern Australia. A study of Australian dogs that included dogs from an area in Sydney that is enzootic for *Ixodes holocyclus* , dogs from people with symptoms consistent with “Lyme disease‐like syndrome,” mostly from coastal New South Wales and Western Australia, dogs used for manufacture of tick antiserum for treatment of tick paralysis by exposure to *I. holocyclus* and dogs from an Indigenous community in Western Australia found no evidence of infection with *B. burgdorferi* , *Ehrlichia* spp., and *Dirofilaria immitis*. One dog had evidence of exposure to *A. platys* [17]. Some vector‐borne infections that have a stronger association with ICGN in dogs such as *B. burgdorferei* and *L. infantum* are not present in Australia or New Zealand [4, 17]. Together, these observations support the likely low prevalence of vector‐borne disease in the population of dogs included in our study, but these dogs were not consistently tested for vector‐borne disease. Interestingly, Australian dogs diagnosed with immune‐mediated hemolytic anemia have a low prevalence of associative immune‐mediated hemolytic anemia, with 13% of dogs having an identified trigger for this disease and only 1.3% having infection as the likely trigger [18]. The higher prevalence of ICGN in countries with more serology positive dogs such as North America and Europe might provide some epidemiological support for an association between infectious disease and ICGN, but ICGN likely represents a heterogeneous group with multiple possible causes. It is also possible that a genetic basis exists for the difference in prevalence of ICGN between Australian and New Zealand and North American and European dog populations.

Glomerulosclerosis was a common cause of proteinuric renal disease in dogs in our study and overseas studies [1, 2]. Glomerulosclerosis was the most common category of non‐ICGN disease (49%) in our study. As observed in our study, dogs with glomerulosclerosis tend to be older, whereas dogs with ICGN are younger than other dogs with glomerular disease [1, 19]. Although a previous study found that 62.3% of dogs were female, 41% of dogs with glomerulosclerosis were female in our study [19].

Clinicopathologic data differed among different types of glomerular disease in dogs in our study, but there was substantial overlap. Given this overlap, renal biopsy remains the most important tool to optimize treatment for dogs with persistent renal proteinuria. Dogs with membranous glomerulonephropathy had lower serum albumin concentrations than other dogs with proteinuric kidney disease in our study. This observation is consistent with other studies in which serum albumin concentration was lower in dogs with amyloidosis, membranous glomerulonephropathy, membranoproliferative or mixed immune‐complex mediated glomerular disease than in dogs with minimal change disease, focal segmental glomerulosclerosis, and juvenile nephropathies [1], reflecting more severe proteinuria. In our study, as in others, dogs with non‐glomerulosclerotic renal lesions had more severe azotemia than dogs with glomerulosclerosis [19]. Other nephropathies in our study included dogs with juvenile nephropathy, interstitial nephritis, and acute tubular injury, which likely accounts for the higher serum creatinine concentrations and lower UPCR in these dogs.

Dogs in our study had some demographic differences compared with dogs undergoing renal biopsy in North America, the United Kingdom, and Europe [1, 2, 3]. The age of included dogs was similar [1, 2], but males outnumbered females in our study in contrast to those in North America, the United Kingdom, and Europe [1, 2, 3]. Golden Retrievers and Labrador Retrievers were uncommon breeds in our study, with these breeds being more common in other studies [1, 2]. Miniature Schnauzers were the most common breed, but they comprised only 2.5% of dogs registered with Dogs Australia, the Australian registration body for purebred dogs, in 2023 [20]. Because only one Miniature Schnauzer (10%) in this study had ICGN and previous reports indicate a lower prevalence of ICGN (22%) in the United States, the higher number of Miniature Schnauzers in our study might have contributed to the lower ICGN prevalence in our study. That Miniature Schnauzers were the most common breed could reflect a genetic predisposition to proteinuric kidney disease such as glomerulosclerosis within the Australian and New Zealand dog population.

Limitations of our study include that renal biopsy samples were available from only 3 of 8 states and territories in Australia, likely reflecting the distribution of referral veterinary hospitals during the time period studied. Because renal biopsy is expensive, selection bias could have been present in the dogs undergoing this procedure. This possibility could be further impacted by the delay in results when renal biopsy samples are submitted because of the distance of Australia and New Zealand to overseas veterinary renal pathology laboratories; some dogs might receive treatment trials rather than renal biopsy.

A limitation of comparing clinicopathological variables between dogs with ICGN and non‐ICGN lesions is that some histological changes could represent a pattern of injury associated with multiple potential causes, rather than a specific disease [21, 22, 23]. As an example, in the classification system currently used in dogs, dogs with focal segmental glomerulosclerosis are included in the non‐ICGN category, whereas primary focal segmental glomerulosclerosis in people is considered the consequence of an immunologic cause related to circulating glomerular permeability factor [22] and Kidney Disease: Improving Global Outcomes guidelines recommend such people be treated with high‐dose immunosuppression [24]. However, at present this relationship is unproven in dogs and requires more detailed investigation of dogs with this specific glomerular lesion.

In summary, dogs with protein‐losing nephropathies in Australia and New Zealand are more likely to have non‐immune complex‐mediated disease than dogs from North America and Europe, potentially because of lower exposure to infectious diseases. Overall, dogs with glomerulosclerosis are older, and dogs with membranous nephropathies have lower serum albumin concentrations than other proteinuric dogs, although substantial overlap exists in clinicopathological features.

## Disclosure

Authors declare no off‐label use of antimicrobials.

## Ethics Statement

Authors declare no institutional animal care and use committee or other approval was needed. Authors declare human ethics approval was not needed.

## Conflicts of Interest

The authors declare no conflicts of interest.
